# Papillary thyroid cancer associated with syndrome of inappropriate antidiuresis: a case report

**DOI:** 10.1186/1752-1947-4-110

**Published:** 2010-04-21

**Authors:** Fabian Beier, Lukas Moleda, Viktoria Guralnik, Philipp Hahn, Katharina Schardt, Reinhard Andreesen, Jürgen Schölmerich, Andreas Schäffler

**Affiliations:** 1 Division of Hematology and Oncology, University Hospital Regensburg, Franz-Josef-Strauß-Allee 11, 93042 Regensburg, Germany; 2 Department of Internal Medicine I, University Hospital Regensburg, Franz-Josef-Strauß-Allee 11, 93042 Regensburg, Germany; 3 Institute of Pathology, University Hospital Regensburg, Franz-Josef-Strauß-Allee 11, 93042 Regensburg, Germany

## Abstract

**Introduction:**

The syndrome of inappropriate antidiuresis is the most common cause of euvolemic hypo-osmolality. This syndrome is associated with a wide variety of diseases. However, its most frequent causes are related to malignancies, especially lung cancer. In this case report, we describe an unknown association of the syndrome of inappropriate antidiuresis with papillary thyroid cancer.

**Case presentation:**

We present the case of a 71-year-old Caucasian, German woman with marked hyponatremia and neurological symptoms. After a detailed clinical investigation, the common causes of syndrome of inappropriate antidiuresis and other malignancies were ruled out. A thyroid nodule was detected by ultrasound and magnetic resonance imaging. Although fine needle aspiration cytology showed negative results, our patient underwent surgery. Papillary thyroid cancer was later diagnosed. After total thyroidectomy, a complete remission of the clinical symptoms occurred and our patient subsequently had iodine-131 radioactive therapy. Hyponatremia was no longer observed during the follow-up investigations.

**Conclusion:**

This is the first reported case of paraneoplastic syndrome of inappropriate antidiuresis caused by papillary thyroid carcinoma. Since its symptoms occurred before the development of local symptoms, total thyroidectomy may provide a timely and efficient treatment for the underlying malignancy.

## Introduction

The first clinical description of a patient with syndrome of inappropriate antidiuresis (SIAD) was published by Schwartz *et al. *in 1957 [1]. Two patients with lung cancer presenting with hyponatremia and continual loss of urinary sodium were described. The authors concluded that the syndrome is caused by inappropriate release of the antidiuretic hormone (ADH). Their hypothesis was later confirmed in several studies [2].

The main clinical findings of SIAD are as follows: (i) hyponatremia with decreased effective serum osmolality (<275 mmol/kg), (ii) clinical euvolemia, (iii) urine osmolality (>100 mmol/kg) above the respective serum osmolality, (iv) a high rate of renal sodium excretion (>40 mmol/L), and (v) normal thyroid and adrenal functions and absence of diuretic agents (Table 1) [3]. The syndrome may be triggered by conditions such as malignant tumors, drugs, pulmonary diseases or central nervous system disorders. The vast majority of patients whose SIAD is caused by malignant disease have bronchogenic carcinoma as the underlying cause. Furthermore, malignancies of the urinary and gastrointestinal tracts, brain tumors, lymphomas, sarcomas, thymomas, oropharynx, and gynecological tumors are typically associated with SIAD [4].

**Table 1:** Overview of the syndrome of inappropriate antidiuresis criteria and our patient's laboratory findings

	SIAD criteria	Patient parameter	Reference values
Serum: Sodium	<135 mmol/L	107 mmol/L	135-150 mmol/L
Serum: Osmolality	<275 mOsm/kg	227 mmol/kg	280-296 mmol/kg
Urine: Sodium	>40 mmol/L	86 mmol/L	170-250 mmol/L
Urine: Osmolality	>100 mOsm/kg	495 mmol/kg	50-1200 mmol/kg
Serum: ADH	(only optional)	6.15 pg/mL*	<6.70 pg/mL

ADH: antidiuretic hormone.

*Inappropriately high in relation to serum osmolality.

Papillary thyroid cancer is the most common subtype of thyroid cancer. The prognosis of patients with papillary thyroid cancer is very good (10-year overall survival in more than 80% of cases) [5]. So far, only a few cases of thyroid cancer have been described in relation to hyponatremia [6]. However, research on the association of papillary thyroid cancer with SIAD has yet to be published.

In this report, we describe a patient with papillary thyroid cancer associated with neurological symptoms resulting from marked hyponatremia due to SIAD. The complete disappearance of her symptoms after total thyroidectomy confirmed the diagnosis of paraneoplastic SIAD related to papillary thyroid cancer.

## Case presentation

In May 2008, a 71-year-old Caucasian, German woman was referred to our hospital presenting with weakness, somnolence, confusion and slurred speech. As reported by her family, her symptoms had slowly deteriorated within the previous three months. On physical examination, our patient had no edema and no evidence of dehydration or hyperhydration. Her initial laboratory evaluation, however, showed severe hyponatremia (107 mmol/L), low serum osmolality (227 mmol/kg), high urine sodium (86 mmol/L), and urine osmolality ranging above the normal serum osmolality (495 mmol/kg) (Table 1). Her renal function was normal (creatinine = 0.48 mg/dL). Although unnecessary for the diagnosis, her ADH level was measured in the serum and found to be inappropriately high in relation to the respective serum osmolality. Cerebral MRI revealed no abnormalities, suggesting that hyponatremia had caused her neurological symptoms. As thyroid parameters and adrenal function tests were normal, endocrine dysfunction could be excluded.

Our patient's medical history revealed intermittent atrial fibrillation and hypertension for which she had been treated with metoprolol and ramipril for more than three years. A drug-induced SIAD was considered as very unlikely due to her long history of taking metoprolol and ramipril. The medication was maintained during her diagnosis and treatment. Her SIAD did not reappear after thyroidectomy. Neither the parameters investigated nor the medication given provided an alternative explanation for the presence of hyponatremia, thus establishing a diagnosis of SIAD.

**Figure 1:**
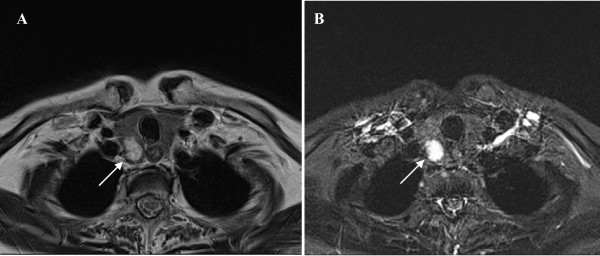
**MRI scan of the neck**. (A) T2 blade sequence. (B) T2 blade sequence with contrast enhancement. The thyroid nodule of the right lobe is indicated by a white arrow. (Images were provided by the Institute of Radiology, University of Regensburg).

Our patient was treated with a fluid restriction of 1.0 to 1.5 liters daily, which led to a continuous increase in her serum sodium levels. This was paralleled by her improved neurological status. A computed tomography (CT) scan of her chest and abdomen failed to detect a tumor mass, while both upper and lower gastrointestinal tract endoscopy yielded negative results. Gynecological investigation also produced no pathological findings. A thyroid nodule of her right lobe was diagnosed by ultrasonography and MRI of her neck (Figure 1). Although the nodule indicated the suspicion of thyroid cancer, a fine needle biopsy did not show malignant cells.

**Figure 2:**
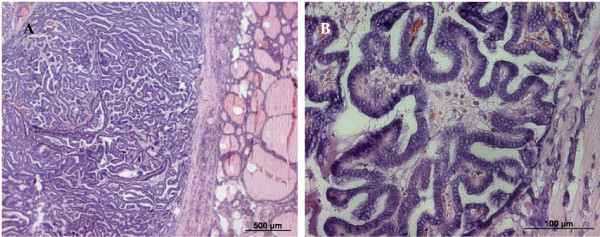
**Histological slides of the resected differentiated papillary thyroid cancer**. (A) Papillary thyroid cancer. (B) Papillary thyroid cancer, high magnification, illustrating the papillary structures.

Since the diagnosis of SIAD suggested the presence of an occult tumor, our patient was referred to the surgery department and underwent total thyroidectomy without lymph node dissection. Her histological examination revealed a 1.5 cm sized differentiated papillary thyroid cancer (pT1 N0 M0) (complete resection with no microscopic residual tumor - RO) (Figure 2). A few days after surgery, she showed stable serum sodium levels without fluid restriction and a complete disappearance of neurological symptoms. Seven weeks after thyroidectomy, she underwent radioactive therapy with 5154MBq iodine-131 (I-131) and out-patient control of her serum sodium. Subsequent osmolality did not show any evidence of SIAD relapse.

## Discussion

Paraneoplastic SIAD has been described in relation to a wide variety of malignant tumors. The syndrome has a prevalence of up to 15% in patients diagnosed with lung cancer, especially small-cell lung cancer [4]. Furthermore, SIAD is present in 3% of patients with head and neck tumors. A detailed analysis of the available literature revealed no association between thyroid cancers and SIAD [7]. In a larger prospective study investigating head and neck tumors, no evidence of the syndrome was observed before and after neck dissection in patients with thyroid cancer [8].

While rare, paraneoplastic syndromes, especially ectopic adrenocorticotropic hormone (ACTH) synthesis, have been described as being related to medullary thyroid cancer, and fewer associations have been reported in cases of papillary thyroid cancer [9]. To the best of our knowledge, cases of paraneoplastic syndromes are sparse in patients suffering from papillary thyroid cancer, such as polymyalgia rheumatica, hypercalcemia, dermatopolymyositis, paraneoplastic neutrophilia, and neurological paraneoplastic syndromes such as myoclonus, optic neuritis and Isaac's Syndrome [10-17].

Symptomatic hyponatremia was reported in five patients with metastatic papillary or follicular thyroid cancer in relation to a low-iodine diet and levothyroxine withdrawal prior to I-131 treatment [6]. All these patients presented with hyponatremia and hypothyroidism. Four of the five patients underwent total thyroidectomy and all developed hyponatremia between surgery and the initiation of I-131 radioactive therapy. Based on this, endocrine dysfunction has to be ruled out before making a diagnosis of SIAD [3].

Surgery (total thyroidectomy) and subsequent radioactive therapy are standard procedures for the treatment and management of patients with papillary thyroid cancer. The surgical treatment is based on thyroidectomy and the excision of locoregional lymph node metastases. I-131 radioactive therapy is usually administered four to 12 weeks after surgery and its purpose is to destroy any remnant of thyroid tissue. Differentiated thyroid cancers, such as papillary thyroid cancer, respond to the stimulation of the thyroid-stimulating hormone (TSH). Based on this, the suppression of TSH levels using supraphysiological doses of levothyroxine is recommended in order to decrease the rate of recurrence. A further part of the long-term management is the control of thyroglobulin levels as a possible marker of recurrence or residual disease [5]. Our patient received the standard treatment according to the official guidelines for total thyroidectomy and post-operative I-131 radioactive therapies.

There are several treatment options for SIAD. Slight hyponatremia can be treated by fluid restriction and by monitoring the sodium levels of patients. In addition, low doses of loop diuretics can be administered in order to increase their serum sodium level. If a patient is suffering from acute neurological symptoms, a hypertonic saline infusion (3% sodium chloride [NaCl]) might be necessary to correct the sodium levels. It is essential to ensure a very slow rise in sodium levels in order to avoid cerebral damage, such as pontine myelinolysis, which can be caused by fast changes. In this context, a so-called "limited and controlled" therapy has been suggested (elevating sodium levels of 0.5 to 1 mmol/L/h to a maximum of 120 mmol/L and not to normal ranges).

Currently, there are no official guidelines for the treatment of SIAD. Every physician has to evaluate the best therapy for treating hyponatremia [3]. In our case, fluid restriction was sufficient to improve our patient's sodium levels.

## Conclusion

The complete disappearance of our patient's symptoms after total thyroidectomy confirmed the diagnosis of paraneoplastic SIAD related to papillary thyroid cancer. This is the first reported case of paraneoplastic SIAD caused by papillary thyroid carcinoma. As SIAD symptoms appeared before the development of local symptoms, papillary thyroid cancer has to be added to the list of tumors associated with SIAD. This knowledge may lead to a timely and efficient treatment for this underlying malignancy.

## Competing interests

The authors declare that they have no competing interests.

## Authors' contributions

FB wrote the manuscript and was involved in the diagnosis and treatment of our patient. LM, VG and PH were involved in the diagnosis and treatment of our patient. RA critically reviewed the manuscript. JS was involved in the diagnosis and treatment of our patient and critically reviewed the manuscript. AS acted as the supervising attending physician of our patient and contributed significantly to the completion of the manuscript. All authors read and approved the final manuscript.

## Consent

Written informed consent was obtained from the patient for publication of this case report and any accompanying images. A copy of the written consent is available for review by the Editor-in-Chief of this journal.
